# A neurochemical closed-loop controller for deep brain stimulation: toward individualized smart neuromodulation therapies

**DOI:** 10.3389/fnins.2014.00169

**Published:** 2014-06-25

**Authors:** Peter J. Grahn, Grant W. Mallory, Obaid U. Khurram, B. Michael Berry, Jan T. Hachmann, Allan J. Bieber, Kevin E. Bennet, Hoon-Ki Min, Su-Youne Chang, Kendall H. Lee, J. L. Lujan

**Affiliations:** ^1^Mayo Clinic College of Medicine, Mayo ClinicRochester, MN, USA; ^2^Department of Neurologic Surgery, Mayo ClinicRochester, MN, USA; ^3^Department of Neurology, Mayo ClinicRochester, MN, USA; ^4^Division of Engineering, Mayo ClinicRochester, MN, USA; ^5^Department of Physiology and Biomedical Engineering, Mayo ClinicRochester, MN, USA

**Keywords:** deep brain stimulation (DBS), feedback control systems, local field potentials (LFP), fast scan cyclic voltammetry (FSCV), machine learning, individualized medicine

## Abstract

Current strategies for optimizing deep brain stimulation (DBS) therapy involve multiple postoperative visits. During each visit, stimulation parameters are adjusted until desired therapeutic effects are achieved and adverse effects are minimized. However, the efficacy of these therapeutic parameters may decline with time due at least in part to disease progression, interactions between the host environment and the electrode, and lead migration. As such, development of closed-loop control systems that can respond to changing neurochemical environments, tailoring DBS therapy to individual patients, is paramount for improving the therapeutic efficacy of DBS. Evidence obtained using electrophysiology and imaging techniques in both animals and humans suggests that DBS works by modulating neural network activity. Recently, animal studies have shown that stimulation-evoked changes in neurotransmitter release that mirror normal physiology are associated with the therapeutic benefits of DBS. Therefore, to fully understand the neurophysiology of DBS and optimize its efficacy, it may be necessary to look beyond conventional electrophysiological analyses and characterize the neurochemical effects of therapeutic and non-therapeutic stimulation. By combining electrochemical monitoring and mathematical modeling techniques, we can potentially replace the trial-and-error process used in clinical programming with deterministic approaches that help attain optimal and stable neurochemical profiles. In this manuscript, we summarize the current understanding of electrophysiological and electrochemical processing for control of neuromodulation therapies. Additionally, we describe a proof-of-principle closed-loop controller that characterizes DBS-evoked dopamine changes to adjust stimulation parameters in a rodent model of DBS. The work described herein represents the initial steps toward achieving a “smart” neuroprosthetic system for treatment of neurologic and psychiatric disorders.

## Introduction

Neurologic and psychiatric disorders can be characterized by motor, behavioral, cognitive, affective, or perceptual traits that affect how individuals move, feel, think, and behave (Benabid et al., 2005; Nemeroff, 2007; Williams and Okun, 2013). These disorders affect over 94 million people in the United States alone with health-care related costs exceeding $648 billion (Logothetis, 2003b; Benabid et al., 2005; Speert et al., 2012; Williams and Okun, 2013). Although most individuals suffering from neurologic and psychiatric disorders are successfully treated with a combination of medications and therapy, up to 30% of patients are unable to respond to standard therapeutic interventions (Olanow et al., 2000; Benabid et al., 2005; Hamani et al., 2006; Nemeroff, 2007; Williams and Okun, 2013). For these treatment-resistant patients, high-frequency electrical stimulation of subcortical brain structures, known as deep brain stimulation (DBS), presents a highly successful therapeutic alternative (Benabid et al., 2005; Williams and Okun, 2013). DBS is FDA-approved for the treatment of Parkinson's disease (PD) and essential tremor (ET) (Benabid et al., 1987, 1991; Burchiel et al., 1999; Koller et al., 2001; Obeso and Guridi, 2001; Simuni et al., 2002; Rehncrona et al., 2003; Germano et al., 2004; Rodriguez-Oroz, 2004; Blomstedt and Hariz, 2010; Moro et al., 2010; Weiss et al., 2013). Additionally, DBS has received humanitarian device exemptions for dystonia and obsessive-compulsive disorder, and there are multiple studies underway for the treatment of other neurologic and psychiatric disorders (Benabid et al., 1987, 1991; Burchiel et al., 1999; Obeso and Guridi, 2001; Simuni et al., 2002; Velasco et al., 2005; Lim et al., 2007; Mueller et al., 2008; Blomstedt and Hariz, 2010; Denys et al., 2010; Fisher et al., 2010; Ramasubbu et al., 2013).

Brain stimulation has been an important tool in the field of neurosurgery pioneered by Spiegel and Wycis (Blomstedt and Hariz, 2010). Intra-operative electrical stimulation of neural tissue has been used since the early days of human stereotaxis to identify surgical targets (Gildenberg, 2003, 2005). Application of brain stimulation in modern-day neurosurgery was revolutionized by Benabid and colleagues, who used high frequency stimulation (typically 100–130 Hz) delivered directly into specific brain regions to mimic the effects of surgical lesions without performing any tissue resection (Benabid et al., 1987, 1991; Blomstedt and Hariz, 2010). DBS achieves therapeutic benefits by delivering electrical currents to specific anatomical targets within the brain via multi-contact electrodes connected to implanted pulse generators. In DBS therapy, a balance between maximal clinical improvement and minimal stimulation-induced side effects is typically achieved by adjusting active electrode contacts, stimulus frequency, amplitude, and pulse duration.

Clinical DBS programming is an iterative process in which stimulation parameters are adjusted in order to maximize therapeutic benefits while minimizing side effects (Morishita et al., 2013) Although many DBS patients require minimal stimulation adjustment following surgery, many more require several months of regular parameter adjustments before optimal therapeutic results can be achieved (Okun et al., 2005; Bronstein et al., 2011; Kluger et al., 2011). However, sustaining these therapeutic benefits requires subsequent adjustment of stimulation parameters every few months (Mayberg et al., 2000, 2005; Deuschl et al., 2006; Moro et al., 2006; Frankemolle et al., 2010; Mure et al., 2011). Therefore, existing clinical programming and stimulation paradigms are poorly suited to cope with the dynamic and comorbid nature of most neurologic disorders. This, in turn, highlights the need for dynamic feedback systems that can continually and automatically adjust stimulation parameters in response to changes within the environment of the brain.

## Therapeutic stimulation paradigms

The therapeutic success of DBS depends not only on accurate surgical targeting and electrode implantation, but also on the ability to optimize stimulation parameters to maximize therapeutic benefits while minimizing side effects. Clinical strategies for therapeutic DBS programming require multiple post-operative visits during which experienced clinicians perform clinical evaluations and corresponding device programming. In each visit, a series of inputs (active contacts, stimulus amplitude, pulse width, and frequency) are adjusted in an attempt to minimize adverse effects while maximizing clinical benefits. Although this strategy has provided significant patient benefit, the results are far from optimal. First, this open loop strategy relies on the subjective experiences of both the patient and clinician, without providing objective feedback to support parameter optimization. Second, the therapeutic response observed in this acute setting does not guarantee sustained therapeutic effects. Disease progression, environmental factors, and behaviorally induced changes in network activity can all render therapeutic stimulation ineffective, requiring additional programming sessions (Obeso and Guridi, 2001; Hunka et al., 2005; Kupsch et al., 2011). Third, the procedure is costly and time consuming. As such, only a fraction of the stimulation parameter space can be practically explored during each session. Fourth, DBS device programming can differ according to the target chosen, the orientation of the electrode relative to the target, the disorder being treated, and the symptoms being treated for a given disorder (Velasco et al., 2007; Ricchi et al., 2012; Min et al., 2013; Miocinovic et al., 2013). Additionally, the timing of programming as well as the waiting time between adjustments can influence when different therapeutic responses can be observed, and these responses also vary between disorders (e.g., Tremor is nearly immediate, whereas depression could take several weeks to observe the effect of a disorder) (Velasco et al., 2007; Ricchi et al., 2012; Min et al., 2013; Miocinovic et al., 2013). Therefore, it is necessary to implement DBS control strategies that can adjust stimulation parameters in real-time according to quantifiable and objective neurochemical, physiological, and behavioral changes while reducing the frequency of clinical interventions. However, before such control strategies can be implemented, it is necessary to improve the understanding of the cellular mechanisms responsible for the network effects of DBS.

The cellular response of single neurons to extracellular electrical fields has been well characterized over short time scales (Smith and Grace, 1992; Benazzouz et al., 2000; Hashimoto et al., 2003; Maurice et al., 2003; Kita et al., 2005; Miocinovic et al., 2006). It is known that excitation of efferent axons or fibers of passage near the site of stimulation results in network changes in neurotransmission and electrical activity (Grill et al., 2004; McIntyre et al., 2004a,b; Johnson et al., 2008; McIntyre and Hahn, 2010; Shah et al., 2010). Furthermore, functional and metabolic imaging studies have shown that successful treatment of neurologic and psychiatric disorders is associated with metabolic normalization in proximal and distal regions of the brain (Mayberg et al., 2000, 2005; Mure et al., 2011). The precise relationships between therapeutic improvement and changes in metabolic patterns remain unknown. As such, current research efforts focus on the use electrophysiology and electrochemistry to elucidate the network effects of DBS (Bledsoe et al., 2009; Lee et al., 2011; Vitek et al., 2012).

## Real-time monitoring of neural activity

Signaling within the brain occurs both electrically and chemically. Technological advances in neural activity monitoring have enabled real-time investigation of cellular and molecular dynamics using electrophysiological and neurochemical probes. While the most used technique involves electrophysiological monitoring of extra-cellular neuronal activity (Smith and Grace, 1992; Benazzouz et al., 2000; Hashimoto et al., 2003; Maurice et al., 2003; Johnson et al., 2005; Kita et al., 2005; Miocinovic et al., 2006) recent advances in electrode technology allow *in vivo* monitoring of synaptic neurotransmitter activity (Roham et al., 2007; van Gompel et al., 2010).

Electrophysiological analysis has been widely used to study stimulation-evoked changes in brain activity, such as increased pallidal (Hashimoto et al., 2003; Kita et al., 2005; Miocinovic et al., 2006) and nigral activity (Smith and Grace, 1992; Benazzouz et al., 2000; Maurice et al., 2003) during subthalamic nucleus (STN) DBS. This has been accomplished by recording single neuron activity (single unit recordings), activity from local groups of neurons (multi unit activity, local field potentials), and distributed signals representing global brain activity [electrocorticograms (ECoGs), electroencephalograms (EEGs)]. Alternatively, neurochemical analysis techniques such as microdialysis, amperometry, and voltammetry, can detect local changes in neurotransmitter concentration evoked by internal and external mechanical, electrical, and chemical stimuli (Dale et al., 2005; Wightman, 2006). Neurochemical recordings have been used to monitor *in vivo* release of analytes such as oxygen, dopamine, adenosine, serotonin, and glutamate in small and large animal models of DBS (Agnesi et al., 2009; Bledsoe et al., 2009; Chang et al., 2009; Kimble et al., 2009; Griessenauer et al., 2010; Shon et al., 2010a,b).

### Single-unit recordings

Single unit recordings capture the activity of distinct neurons *in vivo* by placing a high-impedance microelectrode within the extracellular space surrounding the cell body. These electrodes, having surface areas under 2 × 10-5 cm^2^ (Loffler, 2012), record extracellular potentials representative of intracellular action potentials from neurons adjacent to the electrode tip. The high spatial and temporal resolution provided by single unit recordings allows for precise measurements of neuronal spikes (Buzsáki et al., 2012). However, activity from single units can be difficult to isolate due to crosstalk from neighboring cells (Bai and Wise, 2001). Additionally, single unit recordings can be biased toward activity from larger (e.g., pyramidal) cells (Buzsáki et al., 1983). Furthermore, electrode migration, immune responses (e.g., glial scarring), and disruption of surrounding neural tissue interfere with signal quality and limit reliable single unit activity to acute recording conditions (Carter and Houk, 1993; Polikov et al., 2005).

### Multi-unit recordings

Multi unit recordings capture fast spiking activity from groups of neurons using high-impedance microelectrode arrays. Similar to single unit recordings, this technique provides good spatial and temporal resolution reflecting synaptic events occurring at high frequencies (>800 Hz) (Logothetis, 2003a,b; Mattia et al., 2010). Unfortunately, multi-unit recording arrays suffer from stiff form factors that result in shear-induced inflammation of the surrounding tissue (Cheung, 2007). Furthermore, recording can only occur from the tips of the electrodes, limiting recording selectivity (Maynard et al., 1997).

### Local field potentials

Local field potential (LFP) analysis is an electrophysiological technique for detecting changes in brain activity that offers great potential for understanding the network effects of DBS (Tsang et al., 2012; Priori et al., 2013). This technique is capable of recording chronic electrical activity directly from single and multiple neural units using micro and macro electrodes implanted within the nucleus of interest (Bronte-Stewart et al., 2009; Giannicola et al., 2012). LFPs are typically used to record low-frequency changes in activity across groups of neurons within a volume of interest (Andersen et al., 2004; Buzsáki et al., 2012; Rosa et al., 2012). These activity changes reflect a weighted average of integrative processes and associations between cells that can be detected over longer distances through extracellular space (Logothetis, 2003a,b; Bronte-Stewart et al., 2009). Unfortunately, the longer recording range of LFP techniques is associated with decreased spatial resolution. Despite this limitation, LFP recordings can be performed in real-time using the same DBS electrode, which eliminates the need for additional electrode penetrations (Rossi et al., 2007). Therefore, local field potentials present a good starting point for establishing closed-loop neurostimulation control systems (Rosin et al., 2011; Santaniello et al., 2011; Berényi et al., 2012; Little et al., 2013).

### Global field potentials

Analysis of global brain activity can be used to identify both spontaneous and event-related responses from large groups of neurons. Whole-brain electrophysiological brain activity (i.e., global field potentials) is typically measured using far-field sensors located on the scalp (EEG) or directly on the brain surface (ECoG). These global field potentials can be used to identify information regarding high-level sensory processing, perception, and locomotor activity (Issa and Wang, 2013). For example, EEG signals with low spatial resolution can be recorded non-invasively by non-surgically attaching recording electrodes to the scalp. Alternatively, ECoG signals offer increased spatial resolution, but recording electrodes must be surgically attached at the cortical surface (Buzsáki et al., 2012). Despite the advantages of global field potentials, these signals do not provide insight into activity changes within specific subcortical structures. As such, a system that combines activity analysis within cortical (e.g., EcOG) and subcortical (e.g., LFP) networks should provide a better depiction of network dynamics which, in turn, will be required to develop optimal closed-loop stimulation paradigms (Rosa et al., 2012).

### Neurochemical recordings

Neurochemical sensing allows real-time characterization of neural activity with high spatial resolution and signal specificity (Lee et al., 2004). Microdialysis, amperometry, and voltammetry are three widely used techniques for neurochemical monitoring (Blaha and Phillips, 1996).

Microdialysis is a technique for sampling different analytes and determining their concentration in extracellular fluid (Chefer et al., 2009). This technique offers excellent specificity, selectivity, and sensitivity for quantifying neurotransmitter release in a laboratory setting (Watson et al., 2006). However, it suffers from limited temporal resolution (Smolders et al., 1997; Khan et al., 1999). Therefore, microdialysis is not suitable for real-time clinical application in closed-loop systems.

Amperometry is an alternative technique for measuring analytes in the extracellular space. Amperometric recordings involve the application of a fixed electric potential through a carbon fiber microelectrode (CFM) placed in close proximity to the target cells (Gale et al., 2013; Tye et al., 2013). These CFM are coated with specific enzymes known to react with non-electrolytic analytes of interest, resulting in electroactive products that can be electrically measured (Oldenziel et al., 2004). This allows continuous monitoring of changes in electrical currents within the surrounding extracellular fluid. The detected changes in current are caused by oxidative reactions between the applied potential and analyte molecules within the extracellular space (van Gompel et al., 2010). The downfall of this technique is the high complexity associated with chronic *in vivo* measurements, which require continuous enzyme delivery to detect the breakdown products of the neurotransmitter of interest (Jacobs et al., 2010).

**Figure 1:**
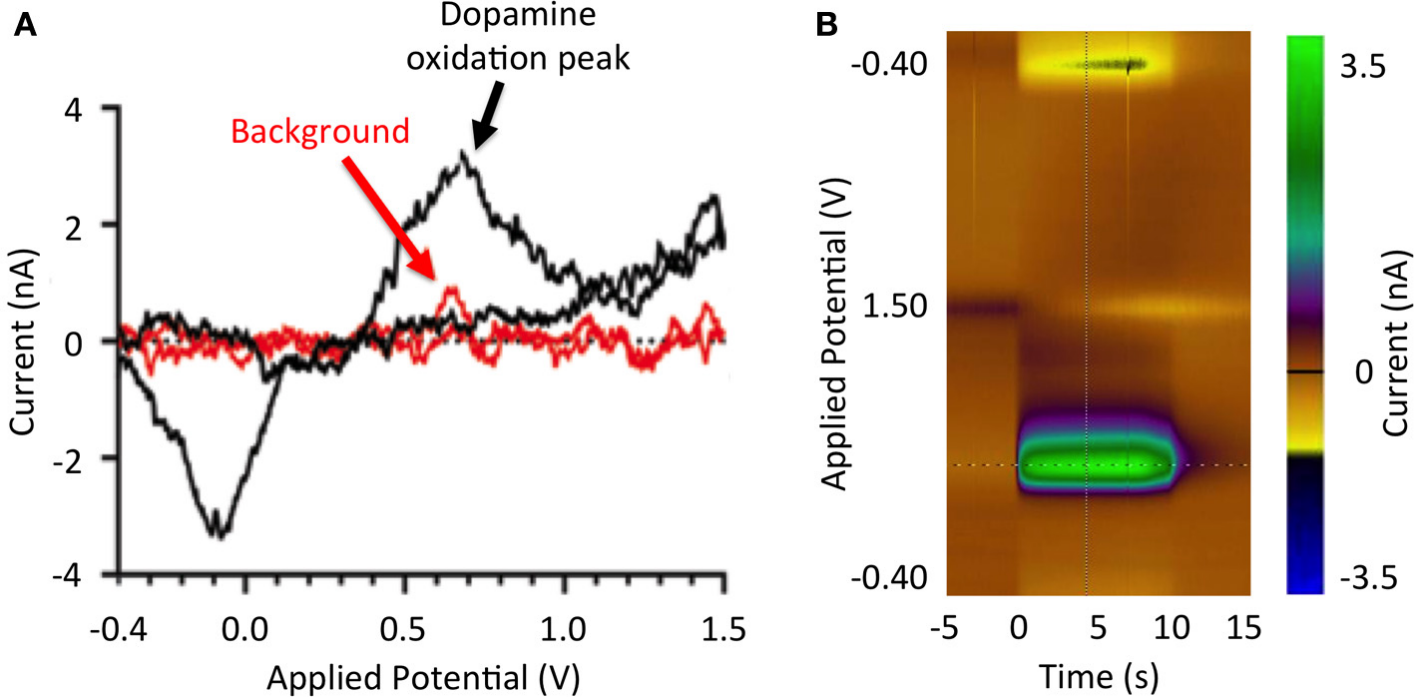
**Stimulation-evoked dopamine responses. (A)** Dopamine redox reactions at the tip of a carbon fiber microelectrode during fast scan cyclic voltammetry. As the potential applied to the electrode increases from −0.4 to 0.0 V, extracellular dopamine is reduced (reduction peak at −3.5 nA). As the applied potential is further increased from 0.0 to 1.0 V, dopamine is oxidized (oxidation peak at 3.5 nA). Measured current background is shown in red. **(B)** Pseudo-color representation of dopamine oxidation current at +0.6 V at DBS onset (100 Hz, 2 ms, 300 μA).

Analogous to amperometry, voltammetry provides real-time high-resolution analyte measurements (Blaha et al., 1990). Specifically, fast scan cyclic voltammetry (FSCV) is a voltammetry technique in which a linearly varying potential is applied to a carbon fiber electrode, allowing for oxidation and reduction of surrounding electroactive molecules to take place (Robinson et al., 2003; Lee et al., 2007). The magnitudes of the analyte oxidation and reduction current peaks are directly proportional to the concentration of analyte oxidized and reduced at the electrode surface (Atcherley et al., 2013). Furthermore, the resulting electrical current vs. applied potential relationships (Figure 1) provide a chemical signature (i.e., voltammogram) that allows identification of specific neurotransmitters or other electroactive analytes (Robinson et al., 2003). FSCV detection of analytes is limited to electroactive molecules such as dopamine, adenosine, and oxygen (van Gompel et al., 2010). Furthermore, the lifetime of CFM is limited to a few months (Kim et al., under review), restricting clinical application of FSCV detection methods to intraoperative approaches.

## Smart DBS control

**Figure 2:**
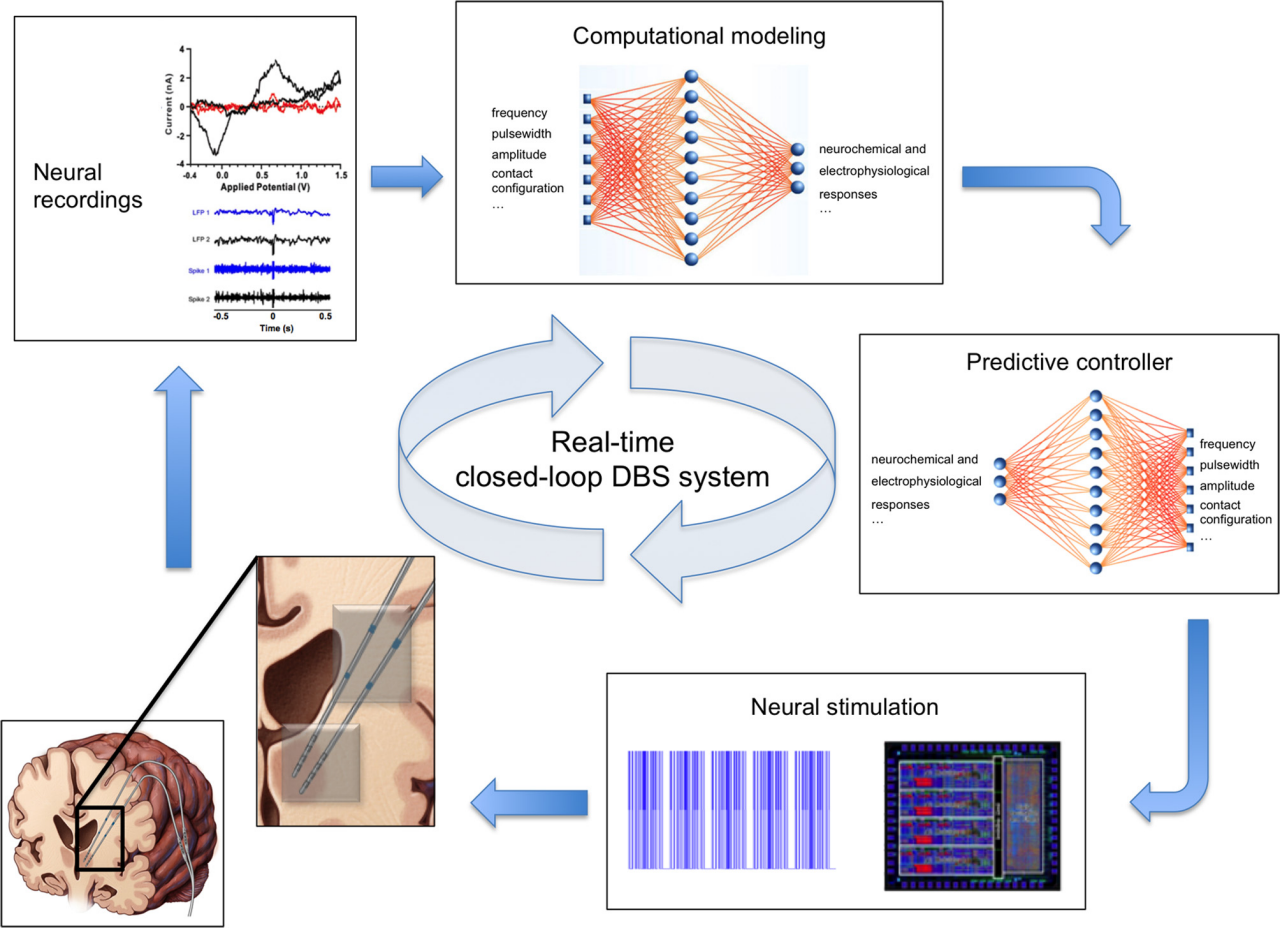
**Real-time closed-loop deep brain stimulation system**. Clockwise from bottom left: (1) Schematic of the human brain with two electrodes (inset) for simultaneous stimulation (gray contacts) and recording of neural activity (blue contacts). (2) Example voltammogram, local field potentials, and single unit activity signals representing recorded neurochemical and electrophysiological neural activity. (3) Computational model of neurochemical and electrophysiological dynamics allows generation and optimization of data beyond the time constraints imposed by experimental conditions. (4) Smart controller uses existing neural activity to predict stimulation parameters required to achieve therapeutic neuromodulation. (5) Predicted stimulation parameters are applied to the brain using an implanted neurostimulation system.

Clinical DBS systems follow an open-loop paradigm. That is, stimulation parameters are pre-programmed into the DBS device and held constant until the next programming session, regardless of the internal state of the system or environmental factors (Foltynie and Hariz, 2010). In contrast, closed-loop DBS systems rely on sensor feedback to monitor the environment and internal state of the system in order to adjust stimulation parameters accordingly (Abbott, 2006; Fagg et al., 2007). That is, stimulation parameters (e.g., stimulation frequency, stimulus amplitude, etc.) are automatically adjusted to maintain specific therapeutic outputs such as tremor suppression in the presence of disturbances, environmental perturbations, and internal network changes (Figure 2). To date, development of closed-loop neuroprosthetic devices has largely focused on using electrophysiological activity as feedback signals (Avestruz et al., 2008; Skarpaas and Morrell, 2009; Rosin et al., 2011; Basu et al., 2013; Grant and Lowery, 2013). Neurochemical-based feedback, however, offers the prospect of finer control of stimulation-induced effects, as it allows activity monitoring from individual types of neurons by virtue of their neurotransmitters. The ability to use neurochemical feedback to control DBS has been demonstrated by characterizing glutamate release using mathematical models linking electrical stimulation to glutamate release in a rat model of DBS (Behrend et al., 2009). Thus, chemical sensing presents a unique opportunity for developing closed-loop smart neurocontrol systems that are optimized for specific disorders and targets, and which can account for intra- and inter-patient variability.

## Neurochemistry of DBS

Studies using small and large animal models suggest that therapeutic DBS coincides with changes in neurotransmitter release (Lee et al., 2004; Shon et al., 2010a,b). It has been established that dopaminergic cell loss in the substantia nigra leads to striatal dopamine deficiency and movement abnormalities in PD patients (MacDonald et al., 2013). It has also been shown that therapeutic STN DBS for treatment of PD decreases the need for exogenous levodopa (Moro et al., 1999; Molinuevo et al., 2000) and has been hypothesized to increase striatal dopamine release (Lee et al., 2009). Complementing findings in electrophysiological and neurochemical sensing studies have shown that STN DBS evokes dopamine release in the striatum of parkinsonian rats (Blaha and Phillips, 1996; Lee et al., 2006). Similarly, stimulation-evoked adenosine release has been recorded intraoperatively in the ventral intermediate nucleus of the thalamus in human patients undergoing DBS for treatment of ET (Chang et al., 2012). However, the specific relationship between DBS and neurochemical activity changes remains unknown. Therefore, understanding the relationships between stimulation parameters and neurotransmitter concentration levels is paramount for developing closed-loop DBS control strategies.

In the following paragraphs, we describe a proof-of-principle approach to closed-loop DBS that automatically adjusts stimulation parameters in order to sustain stable dopamine levels in a rodent model of DBS. The paradigm proposed herein uses FSCV to quantify striatal dopamine release evoked by medial forebrain bundle (MFB) DBS. Additionally, this paradigm relies on non-linear regression, computational modeling, and constrained optimization techniques to parameterize stimulation-evoked dopamine responses. The inverse dynamics of stimulation-evoked dopaminergic responses are modeled using artificial neural networks (ANN), which also predict stimulation parameters required for sustaining target dopaminergic concentration levels. The performance of this closed-loop paradigm was evaluated by comparing target dopaminergic responses to *in vivo* dopaminergic responses achieved using ANN-predicted stimulation parameters (**Figure 4**). While focused on DBS of ascending dopaminergic fibers in the MFB for evoking dopamine release in the rat striatum (Agnesi et al., 2009), this closed-loop paradigm is applicable to a variety of analytes, targets, and neurologic disorders.

### Experimental paradigm

To quantify the dynamics of stimulation-evoked dopamine release, recording FSCV CFM and bipolar DBS macroelectrodes were implanted into the striatum and MFB, respectively, in four anesthetized rats. All animal procedures were performed according to the guidelines of the Mayo Clinic Institutional Animal Care and Use Committee (IACUC). Animals were kept on a standard 12 h light-dark cycle with access to food and water *ad libitum* in conventional housing in accordance with National Institutes of Health (NIH) and US Department of Agriculture guidelines.

Animals were anesthetized and the head was fixed in a Kopf stereotactic frame (David Kopf Instruments, California) for electrode targeting. Following brain exposure, one bipolar stimulating electrode, one FSCV recording electrode, and one silver-chloride reference electrode was inserted into the left MFB, striatum, and contralateral cortex, respectively. Recording electrodes were allowed to stabilize within the tissue environment for 20 min. Finally, the electrodes were connected to a wireless stimulator and neurotransmitter sensor for real-time detection of stimulation-evoked dopamine release (Kimble et al., 2009; Chang et al., 2013).

Following electrode implantation, a comprehensive range of stimulation parameters (Table 1) was used to determine the magnitudes and temporal patterns of stimulation-evoked dopamine release. Stimulation was divided into 65 20-s bins. Each bin corresponded to one combination of stimulation parameters delivered through the active electrode contact. Each stimulation bin was followed by a stabilization and washout period of 180 s.

**Table 1:** **Stimulation parameters**.

**Frequency (Hz)**	**Amplitude (μA)**	**Pulse width (ms)**	**Duration (s)**
60	100–450	2.0	2
100	100–450	2.0	2
20–200	250	2.0	2
20–200	350	2.0	2
60	300	2.0	2
100	300	0.1–2.0	2
60	300	2.0	0.5–8

### Stimulation-evoked neurochemical monitoring

**Figure 3:**
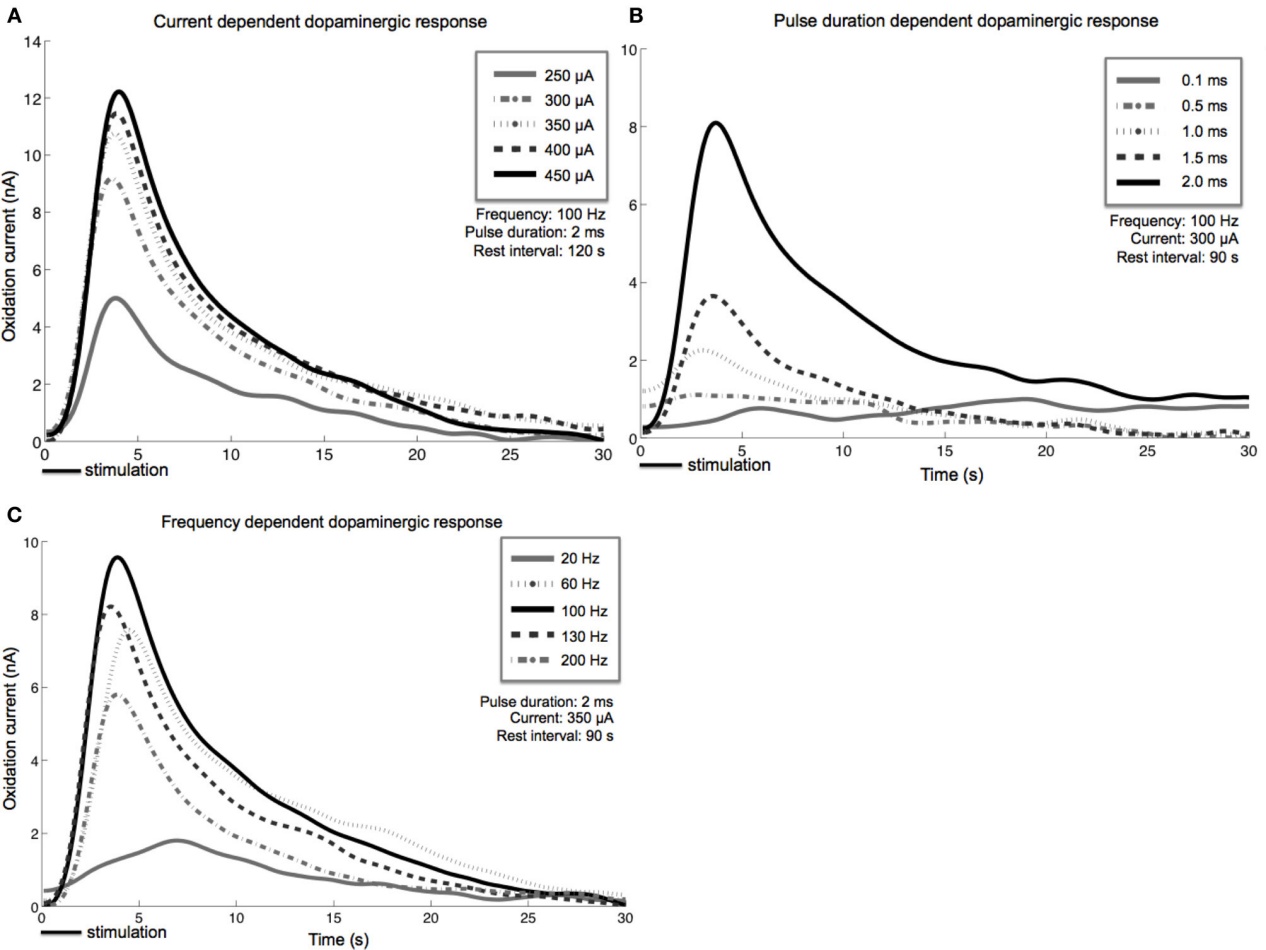
**Stimulation-evoked dopamine release characterization in four anesthetized rats**. A carbon-fiber recording electrode was implanted into the left striatum and a bipolar stimulating electrode was placed within the ipsilateral medial forebrain bundle. A reference silver-chloride electrode was implanted into the contralateral cortex. Current amplitude **(A)**, pulse duration **(B)**, and frequency **(C)** were individually varied while the remaining stimulation parameters were held constant. Stimulus duration was set at 2 s for all experiments described above.

Stimulation-evoked dopamine measurements were obtained by changing the CFM potential from a resting potential of −0.4–1.3 V and back, at a rate of 400 V/s. This triangular waveform was repeated at a frequency of 10 Hz (Chang et al., 2012). The CFM was held at the resting potential between scans. We converted the measured oxidation and reduction current peaks to dopamine concentration using post-operative *in vitro* flow injection analysis calibration of each CFM (Griessenauer et al., 2010). Our preliminary results showed that as MFB DBS amplitude increases, extracellular dopamine levels within the striatum also increase (Figure 3A). A similar response is also observed as pulse duration is increased from 0.1 to 2.0 ms (Figure 3B). Changes in frequency, however, give rise to a different dopaminergic response. Maximum response was observed at 100 Hz, followed by a decrease in dopamine oxidation currents at higher frequencies (Figure 3C).

### Neurochemical response modeling

Implementation of neurochemically-driven closed-loop DBS control strategies requires characterization of the relationship between electrical stimulation and neurochemical responses. To characterize this relationship, stimulation-evoked FSCV dopamine signals were low pass filtered (5th-order Butterworth filter, 100 Hz cutoff frequency) to remove signal noise. Additionally, the responses to individual stimuli were characterized using a combination of 7th-degree polynomial and 2nd-order exponential mathematical models. The mathematical model parameters (eight for the polynomial fit and four for the exponential fit) and corresponding stimuli were presented to a double-layer feedforward ANN with sigmoidal and linear transfer functions (Lujan and Crago, 2009). The hidden layer contained 150-hidden neurons. The inputs to the ANN consisted on the stimulation frequency, pulsewidth, and stimulus amplitude, while system outputs corresponded to the 12 model parameters. Initial weights and biases were selected at random for 10 different initial conditions. Ten corresponding ANNs were trained on 80% of the data (selected at random) using the Levenberg-Marquardt algorithm. The trained ANN with the lowest generalization error, calculated using the remaining 20% of data, was selected as a system model. The resulting system model, when combined with constrained optimization for minimization of stimulation energy, can identify and eliminate mathematical redundancies for the optimal design of the closed-loop controller (Lujan and Crago, 2009).

### Stimulation prediction

In order to provide optimal stimulation, a predictive model that characterizes the inverse relationship between stimulation parameters and dopamine levels was created. Similarly to the system model, the predictive model was created using a double-layer ANN with 600 hidden neurons, as well as sigmoidal and linear transfer functions (Lujan and Crago, 2009). The inputs to the predictive model corresponded to the sets of 12 model parameters, while the outputs corresponded to the three stimulation parameters. This inverse model was then used to predict the stimulation parameters required to sustain specific extracellular dopamine levels within the striatum, thus allowing for feedback control. This was followed by stimulation of the MFB using the predicted parameters, and simultaneous recording of extracellular dopamine levels. Root mean squared (RMS) errors between experimentally measured and desired stimulation-evoked dopamine responses were used to determine controller efficacy. Least-squares regression analysis of the dependencies of actual dopamine levels on target levels was used in an effort to identify systematic (e.g., slope, offset) sources of error (Lujan and Crago, 2009).

### Closed-loop control

**Figure 4:**
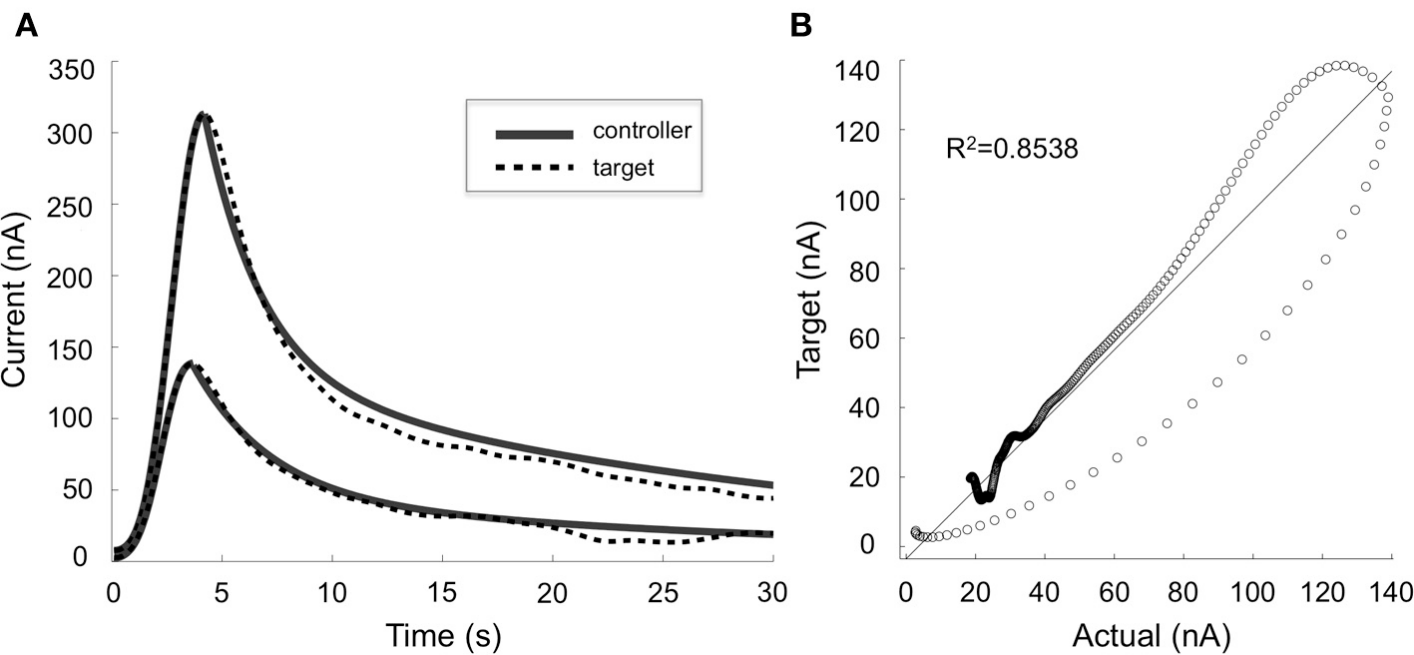
**Controller performance. (A)** Comparison of target (dotted lines) and actual (solid lines) dopaminergic responses evoked by stimulation parameters predicted by the artificial neural network controller. Two typical responses are shown. **(B)** Target and actual responses were compared using linear regression and Pearson's correlation (*R*^2^ = 0.8538).

Our preliminary results in four anesthetized rats suggest that mathematical models can be used to describe the relationships between stimulation-evoked extracellular dopamine responses and DBS parameters (*R*^2^ = 0.8). Furthermore, these results show that adjusting stimulation parameter intensity can modulate dopamine concentration, and that we can use ANN to dynamically predict stimulation parameters required to adjust stimulation-evoked dopamine levels (Figure 4). However, to further understand the network effects of DBS and optimize the therapeutic efficacy of stimulation, it may be necessary to combine electrophysiological (e.g., LFP, ECoG) and neurochemical feedback signals.

## Discussion

Frequent adjustment of stimulation settings has been shown to improve the efficacy of DBS therapy (Rosin et al., 2011), which highlights the nature of the changing brain environment. Thus, a smart, automated system capable of dynamically adjusting stimulation parameters in response to a changing environment becomes critical for improving the therapeutic efficacy of DBS therapy. The proof-of-principle closed-loop DBS system proposed above offers the potential for maintaining therapeutic responses during disease progression. By taking advantage of mathematical models, the paradigm presented here can potentially replace the trial-and-error process currently used in clinical programming with deterministic approaches, thereby achieving optimal therapeutic outcomes while minimizing the number of clinical interventions. In turn, this will ultimately reduce required hospital visits and associated healthcare costs (Fraix et al., 2006).

Before automated adjustment of stimulation parameters can be clinically implemented, however, several key clinical questions need to be investigated. Specifically, the relationship between neurotransmitter levels and symptoms of neurologic disease needs further elucidation. For example, there is indirect evidence to suggest that dopamine depletion plays a role in the symptoms of PD and that dopaminergic medications have a therapeutic response. However, precise concentration changes that occur with symptom exacerbation and amelioration are unknown. Additionally, multiple neurotransmitters may play a critical role in the disease (Fitzgerald, 2014). Thus, optimal neurotransmitters and optimal recording locations should be identified for each disorder. Future work should be directed toward validating closed-loop algorithms, correlating neurotransmitter release to clinical benefit in a large animal disease model of Parkinsonism or ET.

Similarly, an important technical barrier that needs to be addressed is that chronic recordings are not possible using current electrode technology. CFMs are subject to electrode fouling due to the charge imbalance of the waveforms required for FSCV. Efforts are underway to develop electrochemical-sensing techniques capable of extending electrode longevity by renewing the electrochemically active surface following adsorption of chemical species (Takmakov et al., 2010). Additionally, it has been reported that diamond coating may potentially prolong the life of recording electrodes (Roham et al., 2007). Once these technologies have been developed, they will need to undergo extensive safety and efficacy testing and validation in pathological animal models before advancing to clinical trials.

## Conclusions

Conventional neuromodulation systems have been successful at achieving therapeutic outcomes in patients with neurologic and psychiatric disorders. However, limitations in existing technology make ensuring optimal benefits a difficult and expensive endeavor. Correlation of multi-modal electrophysiological and neurochemical recordings may provide new insight into the cellular and molecular mechanisms of therapeutic neuromodulation. Therefore, development of smart DBS controllers that rely on the relationships between neurochemical and electrophysiological recordings with the clinical effects of DBS offers the potential of replacing the trial-and-error process used in clinical programming with a deterministic approach. Furthermore, the versatility and adaptability of such controllers will allow expansion of the clinical indications that can be treated with DBS while tailoring its application to individual patients and symptoms. In turn, these will likely improve clinical outcomes, reduce the time and frequency of patient visits, and lower overall health care costs.

### Conflict of interest statement

J. L. Lujan has intellectual property licensed to Boston Scientific. The authors declare that the research was conducted in the absence of any commercial or financial relationships that could be construed as a potential conflict of interest.
